# Fermentation of Common Nettle Extracts by *Ligilactobacillus salivarius*: New Avenue for the Development of Added-Value Bioactive Products

**DOI:** 10.3390/foods14223905

**Published:** 2025-11-15

**Authors:** Mihajlo Bogdanović, Ana Žugić, Vanja Tadić, Nemanja Krgović, Dragana Mladenović, Aleksandra Djukić-Vuković

**Affiliations:** 1 Faculty of Technology and Metallurgy, University of Belgrade, Karnegijeva 4, 11120 Belgrade, Serbia; 2 Department for Pharmaceutical Research and Development, Institute for Medicinal Plant Research “Dr Josif Pančić”, Tadeuša Košćuška 1, 11000 Belgrade, Serbia; 3 Innovation Center of the Faculty of Technology and Metallurgy, University of Belgrade, Karnegijeva 4, 11120 Belgrade, Serbia

**Keywords:** *Ligilactobacillus salivarius*, common nettle, biotransformation, fermentation, chlorogenic acid, rutin, by-products valorization

## Abstract

The medicinal plants industry generates approximately 30 million tons of by-products annually, most of which remain underutilized. The common nettle (*Urtica dioica* L., *Urticaceae*) is a valuable medicinal plant, rich in polyphenols, vitamins, and essential fatty acids, widely used in food and pharmaceutical applications. Its by-products still lack sustainable valorization strategies. This study aimed to valorize nettle tea by-products and flowers using green extraction techniques and microbial biotransformation. Lyophilized aqueous/ethanolic extracts were fermented with *Ligilactobacillus salivarius* ATCC 11741 to assess whether fermentation could enhance the content and bioavailability of phenolic compounds while maintaining probiotic viability. The results showed that fermentation significantly increased phenolic content and antioxidant activity, with chlorogenic acid concentrations increasing up to 4-fold and caffeic acid derivatives up to 2.5-fold. *L. salivarius* remained viable during fermentation, demonstrating the potential for the production of added-value extracts and probiotic biomass. These findings indicate that nettle by-products can be efficiently converted into functional ingredients through low-energy, environmentally friendly processes, supporting sustainable production and waste valorization.

## 1. Introduction

The medicinal and aromatic plants industry is growing intensively [1]. Due to the abundance of valuable bioactive compounds, the demand for medicinal and aromatic plants rises globally by 10–12% per year [2]. This industry produces about 30 million tons of by-products annually [3]. Inadequate management of these by-products, such as landfilling, can lead to environmental pollution, such as groundwater pollution, greenhouse gas emissions, and the spread of diseases that pose a risk to public health, in addition to wasting significant resources [4,5,6,7]. Necessary steps for the proper management of medicinal and aromatic plants’ by-products entail additional costs for this industry [8]. A cost-effective solution to reducing the impact and costs related to by-products lies in the principles of the circular bioeconomy. It promotes resource reuse and waste elimination, which provides a sustainable solution in line with the UN Sustainable Development Goals [9,10,11].

The common nettle (*Urtica dioica* L., *Urticaceae*) has a significant place in the human diet, food and pharmaceutical industries as a source of nutrients and health-promoting valuable compounds. Nettle is a wild plant found on almost all continents around the world and is widely used in functional food, medicine, and the fiber industries [12,13,14]. This plant has significant phytochemical potential, including polyphenolic compounds, tannins, essential amino acids, and chlorophyll, as well as various minerals and vitamins (A, B, C, E, and K) [14,15,16]. Nettle also contains terpenoids, carotenoids such as β-carotene, violaxanthin, and lycopene [13], and essential fatty acids, such as palmitic acid, cis-9,12-linoleic acid, and α-linolenic acid [17]. The antimicrobial [18], anti-inflammatory [14], anti-diabetic [18], and anti-aging [19] effects of nettle extract have been proven in many studies. These health benefits are linked to a high content of phenolic compounds. Phenolic compounds present in nettle leaf include flavonoids (quercetin, rutin, kaempferol, and isorhamnetin), phenolic acids (caffeoylmalic acid, chlorogenic acid, neochlorogenic acid, caffeic acid), and scopoletin [20,21].

The global nettle root extract market size was around USD 62.3 million in 2024, with an estimated compound annual growth rate of 8.8% to reach USD 145.3 million by the end of 2034 [22]. Nettle root and aerial parts can be valorized through a series of technological operations, including extraction with green solvents and fermentation, employing and emphasizing the principles of the circular bioeconomy.

The biotransformation of herbal extracts by microorganisms is an additional strategy to increase the recovery of bioactive compounds beyond the capabilities of extraction techniques, but it was not sufficiently studied until recently. Extraction can recover only the compounds already present in plant material, while microorganisms can degrade or transform compounds present in substrates and extracts into new chemicals, increasing the concentration of more potent bioactive compounds. The fermentations are most often performed in water-based media, and extractions for final applications in food and pharmaceutical industries favor the use of green solvents, mostly water and ethanol [23]. These extracts could be a suitable substrate for biotransformation after the ethanol evaporation and the inoculation with the desired microorganism.

Lactic acid bacteria (LAB) are particularly good candidates for the biotransformation of plant extract bioactives. LAB play an important role in healthy and functional food and in the pharmaceutical field as probiotics [24,25]. They produce biologically active molecules like exopolysaccharides, bacteriocins, lactic acid, antioxidant enzymes, short-chain fatty acids, etc., which are molecules with health-promoting properties [25]. Exopolysaccharides are associated with anti-ulcer activity, maintenance of the intestinal barrier, anti-biofilm effects against pathogen microorganisms, as well as prebiotic and immunoregulatory properties [26,27,28]. Bacteriocins [29] and lactic acid [30] have antimicrobial properties, while short-chain fatty acids have anti-inflammatory, immunoregulatory, cardioprotective, hepatoprotective, and neuroprotective activities [31,32,33]. LAB can also transform phenolic compounds of plant origin into forms with higher bioavailability [34,35]. Probiotic bacteria from the former genus *Lactobacillus* [36] are often used in the production of fermented foods [37], and their esterases, decarboxylases, reductases, etc., are responsible for the number of transformations reported in the literature.

Additionally, recent discoveries about the intricate interplay of food intake and gut microbiota are opening space for new approaches for the production and utilization of antioxidants. Fermented medicinal plant extracts with probiotic bacteria could be natural alternatives to synthetic antioxidants. They can extend food shelf life and boost the nutritional value of food through bioactive compounds and micronutrients, but at the same time support the gut microbiota.

This work examined the production of a new class of fermented extracts of common nettle tea industry by-products as a source of antioxidants. *Ligilactobacillus salivarius* ATCC 11741 was used for the first time for the fermentation of nettle extracts, as far as it is known to the authors. The kinetics of microbial growth and biotransformation of polyphenols were investigated to test the hypothesis that *L. salivarius* can metabolize and increase the concentration of specific polyphenols in nettle extracts. This strain was chosen because it is part of the human oral microbiota and is widely used as an intraoral probiotic, indicating its potential for the biotransformation of complex plant compounds, and was selected based on preliminary studies (unpublished data). In this way, fermented extracts rich in bioactive compounds and fortified with viable *L. salivarius* can be produced as synbiotics, or as postbiotics if the *L. salivarius* biomass is removed, allowing various applications in food, dietary supplements, and cosmetics.

## 2. Materials and Methods

### 2.1. Fermentation of Extracts

Dry biomass is obtained from the herbal tea industry (“Adonis d.o.o.”, Sokobanja, Serbia). Common nettle flower tea (moisture content 8.75 ± 0.02%) and common nettle by-products consisting of a mixture of flowers, leaves, and stems remaining after the fabrication of filter tea bags, in the form of pulvis (moisture content 8.83 ± 0.04%), were used for further extraction. Extracts of common nettle flowers (FE) and common nettle by-products (BE) were prepared according to the Taguchi-optimized protocol for achieving the highest total phenolic content for specific raw material, as previously reported by Mladenović et al. [38]. Briefly, for the extraction, 3 g of powdered biomass were mixed with 40 mL of 50% ethanol, and the samples were incubated (FE at 25 °C; BE at 37 °C) in an orbital shaker (170 rpm) for 24 h. After incubation, ultrasound-assisted extraction was carried out in an ultrasound bath (90 W) for 10 min. Further, the samples were centrifuged (6000 rpm, 10 min) and filtered to separate the supernatant from the solid. The FE and BE extracts were lyophilized and stored until fermentation. Although the extraction temperatures differed, each was optimized [38] to obtain the maximum amount of total phenolics from the specific raw material, which was taken into account when comparing the extracts.

For fermentation, the extracts were dissolved in sterile distilled water at a concentration of 10 mg/mL. Overnight culture of *L. salivarius* ATCC 11741 (9 log CFU/mL) was centrifuged (12,000 rpm/10 min), the supernatant was removed, and the cells were resuspended in the same volume of the appropriate extract. The extracts were then inoculated with 5% (*v*/*v*) of the resuspended overnight culture to achieve an initial bacterial concentration of 5 log CFU/mL in the media. Fermentation was carried out for 48 h at 37 °C in static microaerophilic conditions. Antioxidant activity, total phenolic content (TPC), pH, sugar concentration, lactic acid concentration, and the number of viable cells were determined. Except for viable cell counting, all tests were performed using the cell-free supernatant obtained after microbiological filtration (0.22 µm). To eliminate the contribution of *L. salivarius* metabolism and biomass to antioxidant activity unrelated to the extract fermentation, the values for inoculated sterile distilled water were subtracted from those of the inoculated extracts. Unfermented extracts, incubated under the same conditions, were used as a control. The kinetics of biotransformation were analyzed by HPLC at the same time intervals (0 h, 7 h, 19 h, 24 h, and 48 h) in both fermented and control samples. The following sample notation was adopted: f-FE—fermented common nettle flower extract; FE—unfermented common nettle flower extract; f-BE—fermented common nettle by-product extract; BE—unfermented common nettle by-product extract.

### 2.2. Evaluation of Viable Cell Number

The number of viable *L. salivarius* cells was determined using Koch’s layered plate method [39]. Pure MRS (de Man, Rogosa, and Sharpe) media inoculated with *L. salivarius* under the same conditions were used as controls. Samples were plated at 0 h, 7 h, 19 h, 24 h, and 48 h of fermentation.

### 2.3. Evaluation of Lactic Acid and Reducing Sugar Concentration in Extracts

The lactic acid was determined by a standard enzymatic method (L-lactic acid assay, Megazyme, Wicklow, Ireland). The method is based on the oxidation of L-(+) or D-(-) lactic acid to pyruvate by the corresponding dehydrogenase in the presence of NAD^+^, resulting in the formation of NADH. The produced NADH, proportional to the lactic acid concentration, was quantified spectrophotometrically at 340 nm after pyruvate was converted to D-alanine and 2-oxoglutarate by D-glutamate–pyruvate transaminase.

The concentration of reducing sugars was determined using a modified 3, 5-dinitrosalicylic acid (DNS) method. Briefly, 200 µL of the sample was mixed with 200 µL of DNS reagent, the reaction mixture was incubated at 98 °C for 10 min, and the absorbance was measured at 540 nm [40]. Sugar concentration was calculated using a glucose standard curve.

### 2.4. Antioxidative Activity Assays

#### 2.4.1. DPPH Assay

The 2,2-diphenyl-1-picrylhydrazyl (DPPH) radical scavenging spectrophotometric assay was performed according to Brand-Williams et al. [41]. DPPH antioxidant activity was calculated using a Trolox standard curve and represented as Trolox equivalents per gram of dry matter (mM TE/g d.m.).

#### 2.4.2. ABTS Assay

The 2,2′-azino-bis(3-ethylbenzothiazoline-6-sulfonic acid) (ABTS) radical scavenging spectrophotometric assay was performed according to Re et al. [42]. ABTS antioxidant activity was calculated using a Trolox standard curve and represented as Trolox equivalents per gram of dry matter (mM TE/g d.m.).

Antioxidant activity (both DPPH and ABTS) was estimated using the following formula:

DPPH or ABTS (%) = ((A_B_ − A_S_)/A_B_) × 100 (1)

A_B_ is the absorbance of the blank (absorbance of solvent—sterile distilled water); A_S_ is the absorbance of the sample.

### 2.5. Total Polyphenol Content (TPC)

The TPC test was performed according to the modified method described by Swain and Hillis [43]. Briefly, 50 μL of the extract was mixed with 250 μL Folin–Ciocalteu reagent and 3 mL of distilled water, followed by vortexing. After that, 1 mL of 15% sodium carbonate solution was added, then vortexed and diluted with distilled water to 5 mL. After 2 h, the absorbance was measured at 765 nm relative to the blank. TPC was calculated using a gallic acid standard curve and presented as gallic acid equivalents per gram of dry matter (mM GAE/g d.m.)

### 2.6. HPLC Method

For qualitative and quantitative analysis of polyphenolic compounds in common nettle extracts, an Agilent 1200 HPLC system equipped with a photodiode-array (PDA) detector and Lichrospher 100RP 18e column (250 × 4.6 mm; 5.0 μm particle size) was used. Mobile phase A was a 0.1 M phosphoric acid solution, while mobile phase B was acetonitrile. Total run time was 70 min using the following gradient program: 11–25% B (35 min), 25–40% B (20 min), 40–65% B (5 min), 65–100% B (10 min). The flow rate was set to 1.0 mL/min, the injection volume was 10 μL, the column temperature was 25 °C, while the PDA detector was set at 260, 280, and 325 nm. All investigated extracts were diluted with deionized water to achieve a concentration of 5 mg/mL, and, before injection, were filtered using a PTFE membrane filter. The identification of compounds was based on the comparison of UV-VIS spectra and retention times with HPLC standards. Once spectra matching succeeded, results were confirmed by spiking with respective standards to achieve a complete identification using the so-called peak purity test. The peaks that did not fulfill these requirements were not quantified. Quantification was performed using the external standards method. The concentrations of standards were as follows: 0.45 mg/mL for chlorogenic acid, 0.48 mg/mL for rutin, 0.42 mg/mL for caffeic acid, and 0.63 mg/mL for *p*-coumaric acid. The results were expressed as mg/g of dried drug weight [44].

### 2.7. Evaluation of Protein Concentration in Extracts and Alpha amino Nitrogen

The protein concentration in extracts was determined according to the modified Lowry method [45]. Reagent A was prepared by mixing 0.2 g of C_4_H_12_KNaO × H_2_O and 10 g of Na_2_CO_3_ with 50 mL of 1 M NaOH, and the solution was made up to 100 mL with distilled water. Reagent B was prepared by mixing 0.2 g of C_4_H_12_KNaO × H_2_O and 1 g of CuSO_4_ with 90 mL of distilled water, and the solution was made up to 100 mL with 1 M NaOH. Reagent C was a 1:15 dilution of Folin–Ciocalteu reagent with distilled water. For the analysis, 200 µL of the sample was mixed with 180 µL of Reagent A, then mixed and incubated for 10 min at 50 °C. Reagent B was added, and the mixture was vortexed intensively for 10 min. Subsequently, 600 µL of reagent C was added, followed by incubation for 10 min at 50 °C. Absorbance was measured at 650 nm. Protein concentration was determined using a bovine serum albumin (BSA) standard curve.

The α-amino nitrogen content was determined by a ninhydrin-based spectrophotometric method [46]. Reagent A was prepared by dissolving 10 g of Na_2_HPO_4_ × 12H_2_O, 0.5 g of ninhydrin, and 0.3 g of fructose (C_6_H_12_O_6_) in distilled water and making up the volume to 100 mL (pH of the final solution was 6.6–6.8). Reagent B was prepared by dissolving 2 g of KIO_3_ in 1 l of 40% ethanol. Glycine was used as a standard. Diluted sample (2 mL) was mixed with 1 mL of Reagent A, heated for 16 min in a boiling water bath, and incubated for 20 min at 20 °C. After cooling, 5 mL of Reagent B was added, and the absorbance was measured at 570 nm relative to a blank (sterile distilled water).

α-amino nitrogen (%) = (A_S_/A_G_) × 2 × R (2)

A_G_ is the absorbance of the glycine standard; As is the the absorbance of the sample; R is the dilution factor.

### 2.8. Statistical Analysis

The studies were carried out in biological duplicates, with each biological replicate measured in technical duplicates. Data analysis was performed using OriginPro^®^ 2024 software (Northampton, MA, USA). All results are shown as means with standard deviations indicated by error bars. One-way and two-way ANOVA were performed for statistical analysis, followed by the Tukey test for mean differences. Differences were considered statistically significant at *p* < 0.05.

## 3. Results and Discussion

### 3.1. Fermentation Parameters

The success of biotransformation depends on how well the substrate is matched to the nutritional needs of microorganisms. This is especially important for probiotics and health-promoting microorganisms, which could be part of gut microbiota or functional food microbial consortia. The growth of *L. salivarius* was investigated in FE and BE, as a valuable source of antioxidants with potential prebiotic ability. In Table 1, characteristics of both inoculated (f-FE, f-BE) and uninoculated (FE, BE) extracts before incubation are shown.

**Figure 1:**
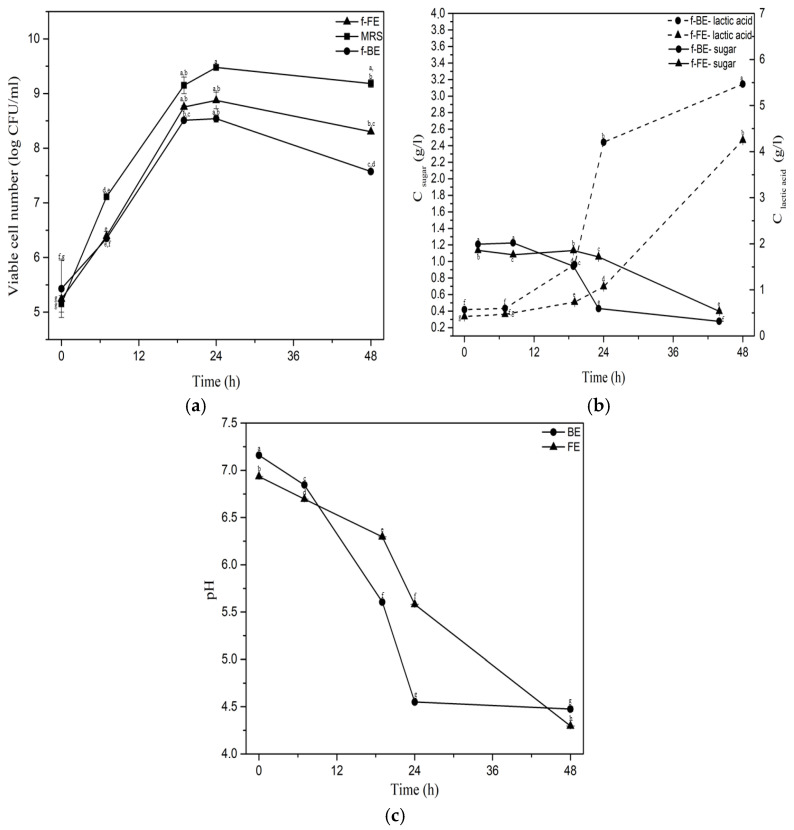
Fermentation parameters: (**a**) viable cell number of *L. salivarius*, (**b**) sugar (solid line) and lactic acid (dashed line) concentrations, and (**c**) pH. Symbols: f-FE (fermented common nettle flower extract) (▲), f-BE (fermented common nettle by-product extract) (●), and MRS (■). Different letters indicate significant differences (*p* < 0.05) among all samples, as determined by two-way ANOVA followed by Tukey’s post hoc test.

The kinetics of nettle extract fermentations by *L. salivarius* on different parameters are shown in Figure 1.

The highest viable cell number of *L. salivarius* was obtained after 24 h of fermentation (Figure 1a). Almost the same viable cell number was in f-FE (8.87 log CFU/mL) and f-BE (8.54 log CFU/mL), and one log unit lower than in MRS. This is a very high number of viable cells considering that extracts have lower sugar concentration, originating only from plant material, in comparison to MRS, which are carbon and nitrogen-rich media, with a reducing sugar concentration of 20 g/L and a high content of easily assimilable nitrogen in the form of peptides and proteins [47]. The observed growth of *L. salivarius* implies the probable prebiotic activity of polyphenols expected to be present in the nettle extracts [48,49].

The lactic acid concentration was the highest after 48 h of fermentation in both f-FE and f-BE, although f-BE (5.46 g/L) reached a higher concentration compared to f-FE (4.24 g/L) (Figure 1b). Lactic acid production reached its full potential at an earlier stage of fermentation in f-BE than in f-FE. Namely, after 24 h of fermentation, lactic acid concentration in f-BE was almost four times higher than in f-FE, suggesting that the composition of f-BE might favor lactic acid production more effectively. A decrease in sugar concentration can be observed as *L. salivarius* metabolizes sugar into lactic acid. Homofermentative bacteria theoretically produce 2 mol of lactic acid from 1 mol of glucose, implying that 1 g of sugar can theoretically yield about 1 g of lactic acid. However, in both extracts (Figure 1b), lactic acid concentration was significantly higher than the initial reducing sugar concentration. Despite *L. salivarius* ATCC 11741 being considered a homofermentative bacterium, this suggests that it utilizes additional extract components, such as polyphenols or other sugars, for lactic acid production. Facultative heterofermentative behavior has been observed in other *L. salivarius* strains [50], and remains to be examined in detail for *L. salivarius* ATCC 11741 in the future.

The pH of the fermented extracts steadily declined during the fermentation (Figure 1c), following a trend observed for lactic acid production (Figure 1b). It has been shown that nettle extracts had a neutral initial pH value, which was maintained above 5 during the first 24 h of fermentation, which is in the optimal pH range for *L. salivarius* growth (5.5 to 6.5) [51]. It has been shown that nettle extracts are rich in alkaline calcium and potassium. For example, ultrasound-obtained nettle extract contains 288.88 mg/L of calcium [52] which may be speculated to partially neutralize the generated lactic acid. 

Lactic acid fermentation of nettle extracts showed that, besides reducing sugars, other compounds could be utilized by *L. salivarius*, supporting growth and lactic acid production. Among the best candidates for biotransformation, in this case, are polyphenols. Hou et al. [53] investigated the fermentation of lychee pulp as a polyphenol-rich substrate by *Lactobacillus plantarum* GDMCC 1.140, *Lacticaseibacillus rhamnosus* GDMCC 1.1798, and by a mixed culture of those two microorganisms for 48 h. The results showed that during the fermentation, the concentration of titratable acids increased, with a simultaneous decrease in pH values in the optimal range for microbial growth, accompanied by a high number of viable cells, and they reported biotransformation of polyphenols. The same trends related to lactic acid production, growth, and pH, were observed in our study on nettle extracts. The impact of fermentation on antioxidant activity and polyphenols was examined further.

### 3.2. Antioxidant Activity and TPC During the Fermentation of Nettle Extracts by L. salivarius

**Figure 2:**
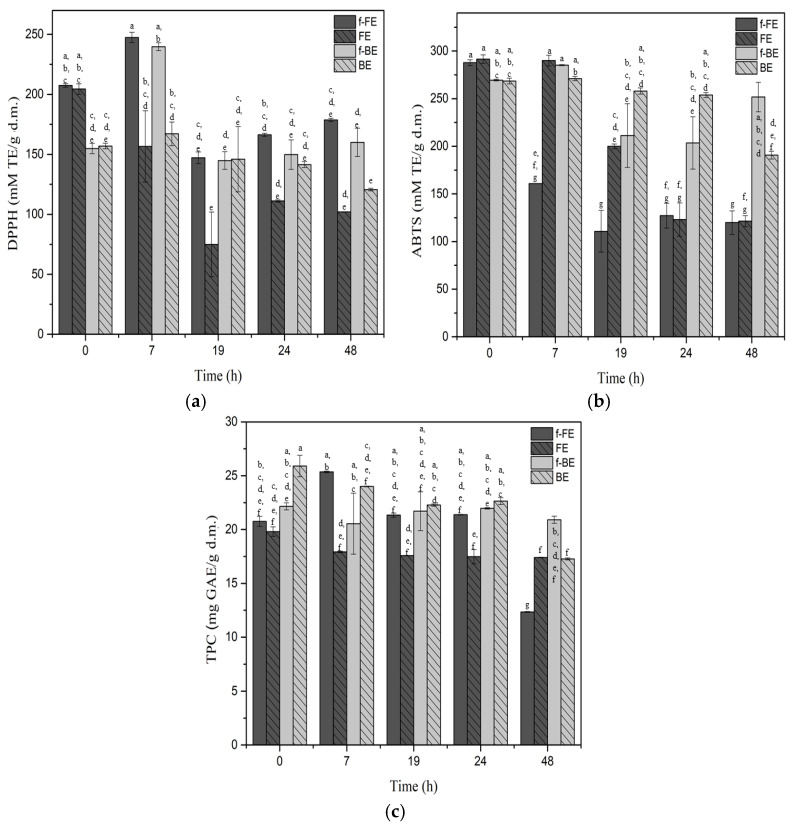
The antioxidant activity of fermented FE and BE determined by (**a**) DPPH, (**b**) ABTS, and (**c**) TPC. Symbols: f-FE (fermented common nettle flower extract—dark gray), FE control (unfermented common nettle flower extract—dark gray patterned), f-BE (fermented common nettle by-product extract—light gray), and BE control (unfermented nettle by-product extract—light gray patterned). Different letters above the bars indicate significant differences (*p* < 0.05) among all samples, as determined by two-way ANOVA followed by Tukey’s post hoc test.

Nettle is an edible plant and part of the human diet, which makes it a very suitable substrate for the assessment of microbial biotransformation by Generally Recognized As Safe (GRAS) microorganisms. We investigated the biotransformation kinetics of significant antioxidant compounds by *L. salivarius* during 48 h of fermentation in f-FE and f-BE. Figure 2 shows the impact of fermentation on (a) DPPH, (b) ABTS, and (c) TPC.

The highest DPPH was after 7 h of fermentation, and with prolonged fermentation, DPPH activity decreased in both f-FE and f-BE extracts, suggesting degradation or biotransformation of antioxidant compounds detected by DPPH (Figure 2a). However, both fermented extracts showed higher DPPH activity over respective controls during fermentation, highlighting the potential of fermentation to enhance the antioxidant activity of nettle extracts. The highest increase over respective controls of 36.67% for f-FE and 30.26% for f-BE was observed after 7 h of fermentation. The by-products are considered lower quality feedstock, but the results showed almost the same DPPH for both fermented extracts after 7 h of fermentation, which was about 240 mM TE/g d.m. In the literature, DPPH for nettle extracts ranges from IC_50_ 88.33 µg/mL of dry matter [54] to 230 mg /mL of dry matter [55], depending on the solvent and extraction technique applied. The top-performing fermented extract (f-FE) in this study demonstrated a calculated IC_50_ of 9.5 mg/mL of dry matter, which can be considered a significant value compared with the literature.

The antioxidant ABTS activity of the f-BE and even the control BE sample showed higher and more stable activity than nettle flower extracts. The f-FE showed a significantly lower ABTS activity during the first 24 h of fermentation, compared to the control, while f-BE had similar or higher ABTS activity than controls (Figure 2b). Fermentation for 48 h improved the ABTS antioxidant potential of nettle residues (f-BE), leading to higher values than those of any other tested sample. This proves the potential of fermentation in the valorization of residues. After 7 h of fermentation, the ABTS in f-BE was 43.64% higher than in f-FE, which emphasizes the superiority of f-BE against the ABTS radical. The highest ABTS activity of 285.24 mM TE/g d.m. was observed in f-BE. The ABTS of f-BE was 23.9 times higher than the value reported for the 50% ethanol nettle extract, reported by Carvalho et al. [56]. In our study, fermentation of extracts obtained from nettle by-products upgraded their antioxidant activity and value. On the contrary, in flower extracts, fermentation negatively affected ABTS activity. This is the opposite trend to that observed for DPPH activity and TPC in f-FE. Fermentation positively contributed more to DPPH activity and TPC in extracts from nettle flowers (f-FE) than from by-products (f-BE) (Figure 2).

Both extracts and controls maintained fairly similar TPC values during the first 24 h of fermentation (Figure 2c). Extended fermentation beyond 7 h did not lead to a significant increase in TPC and showed no clear additional benefit with prolonged fermentation. The highest TPC of 25.35 mg GAE/g d.m. was obtained after 7 h of fermentation in f-FE, which was 13.21% higher than in f-BE in the same time interval. Much lower values for 50% methanol (9.1 mg GAE/g d.m.), 50% ethanol (7.4 mg GAE/g d.m.), and aqueous (7.3 mg GAE/g d.m.) nettle extracts were reported by Vajić et al. [57].

Based on the DPPH, ABTS, and TPC results, 7 h of fermentation can be recommended as the most effective for maximizing the concentration of antioxidant compounds. Long incubation, for 48 h, also negatively affects ABTS and DPPH in controls; therefore, longer fermentations cannot be recommended under the studied conditions. The ABTS assay is more sensitive to hydrophilic antioxidants, whereas the DPPH assay primarily detects lipophilic antioxidants [58]. The observed increase in DPPH and ABTS activities in fermentation may be attributed to the biotransformation of antioxidant compounds, which alters their polarity and affects their solubility, or their release from bonded forms, explaining the increase in antioxidant activities. Overall, these results highlight lactic acid fermentation as a promising strategy to enhance the bioactivity of nettle extracts.

### 3.3. Phenolic Compound Content During Fermentation by L. salivarius

**Figure 3:**
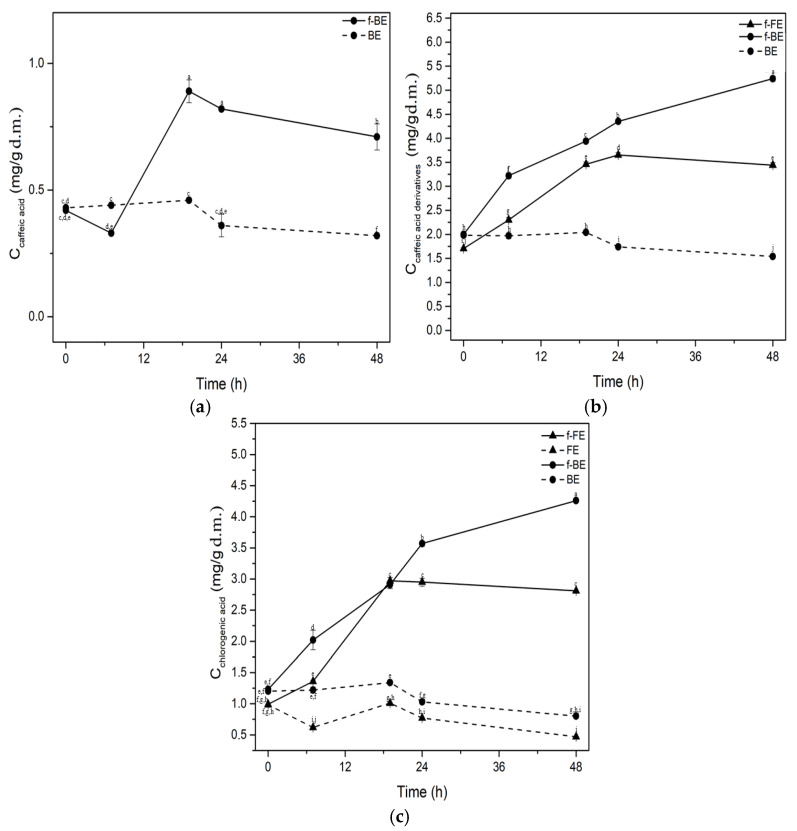
Impact of fermentation on the concentrations of (**a**) caffeic acid, (**b**) caffeic acid derivatives, and (**c**) chlorogenic acid. Symbols: f-FE (fermented common nettle flower extract) (▲, solid line), FE control (unfermented common nettle flower extract) (▲, dashed line), f-BE (fermented common nettle by-product extract) (●, solid line), and BE control (unfermented common nettle by-product extract) (●, dashed line). Different letters indicate significant differences (*p* < 0.05) among all samples, as determined by two-way ANOVA followed by Tukey’s post hoc test.

**Figure 4:**
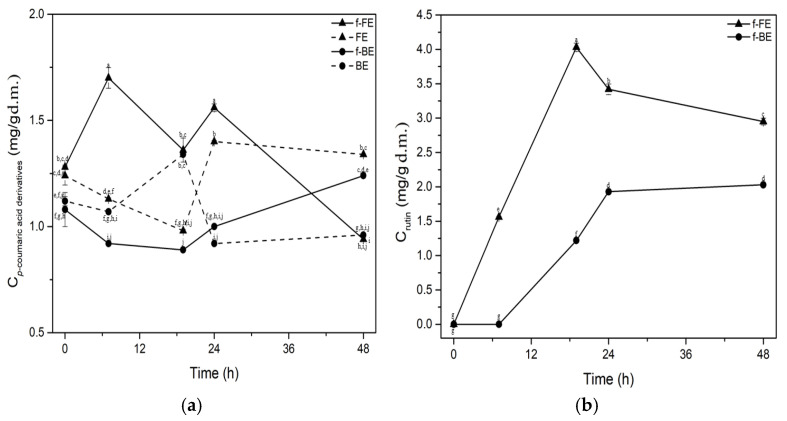
Impact of fermentation on concentrations of (**a**) *p*-coumaric acid derivatives and (**b**) rutin. Symbols: f-FE (fermented common nettle flower extract) (▲, solid line), FE control (unfermented common nettle flower extract) (▲, dashed line), f-BE (fermented common nettle by-product extract) (●, solid line), and BE control (unfermented common nettle by-product extract) (●, dashed line). Different letters indicate significant differences (*p* < 0.05) among all samples, as determined by two-way ANOVA followed by Tukey’s post hoc test.

To gain an insight into the significant changes and biotransformation, the concentrations of phenolic compounds during the fermentation of common nettle extracts are shown in Figure 3 and Figure 4.

Fermentation led to significantly higher concentrations of phenolic compounds regardless of the feedstock extracts, being FE or BE. The concentration of caffeic acid increased after 19 h of fermentation of f-BE by 48.31% compared to the control and remained almost constant until the end of fermentation (Figure 3a). Caffeic acid was not detected in f-FE and FE. Fermentation had a positive influence on increasing the concentrations of chlorogenic acid and other caffeic acid derivatives in both f-FE and f-BE (Figure 3b,c). Caffeic acid derivatives, besides chlorogenic acid, include phaselic acid (caffeoyl-L-malic acid), chicoric acid (2,3-dicaffeoyl-L-tartaric acid), cynarin (1,5-dicaffeoylquinic acid), etc. [59]. During fermentation, f-BE had significantly higher concentrations of caffeic acid derivatives and chlorogenic acid than f-FE. The most significant increase in concentrations was observed up to 24 h of fermentation in both f-FE and f-BE (Figure 3), which implies biotransformation potential during the exponential growth phase of microbial metabolism. At 24 h of fermentation in f-BE, the concentrations of chlorogenic acid and caffeic acid derivatives expressed as caffeic acid were 3.57 mg/g d.m. and 0.82 mg/g d.m., respectively. Fermentation significantly increased the concentration of phenolic compounds in f-BE, reaching values almost equal to the chlorogenic acid and caffeic acid reported by Orčić et al. [60] in 80% methanol nettle extract, while only green solvents were used in our study. Although aqueous extracts are considered less stable and susceptible to contamination in general, fermentation by LAB results in acidification, which prevents contamination and extends shelf life. This could be an important advantage over extracts obtained with conventional solvents. The increase in the concentration of chlorogenic acid due to lactic acid fermentation has been reported in the literature previously [61,62] and likely involves decomposition and release from their bound forms. The relatively low increase in caffeic acid concentration in f-BE during the fermentation was noticed, but part of the available caffeic acid could also be used by *L. salivarius* as a substrate for biotransformation, leading to the higher concentration of caffeic acid derivatives, a trend noticed in f-BE samples (Figure 3a).

The concentration of *p*-coumaric acid derivatives was significantly higher than the control during the first 24 h in f-FE, but decreased later in fermentation. In f-BE, the concentration of *p*-coumaric acid derivatives increased steadily, starting lower than the control and surpassing it after 24 h of fermentation. The highest concentration of *p*-coumaric acid derivatives was observed at 24 h in f-FE, reaching 1.4 mg/g d.m., which is 35.9% higher than the concentration in f-BE (Figure 4a). A positive effect of fermentation on the concentration of rutin in both f-FE and f-BE was also observed (Figure 4b). Rutin was not detected in unfermented extracts. The highest concentration of rutin was observed in f-FE (4.03 mg/d.m.) after 19 h of fermentation. Fermented extracts were shown to have significantly higher concentrations of rutin than the extracts reported by Zeković et al. [63]. They found the rutin yields of 578.36 µg/g d.m., 722.83 µg/g, and 215.49 µg/g d.m., in aqueous nettle extracts obtained through ultrasonic, microwave, and subcritical extraction, respectively. Top-performing fermented extract (f-FE) tested in our study had 6.9, 5.5, and 18.7 times higher concentrations of rutin in extracts in comparison to those obtained by ultrasonic, microwave, and subcritical extraction, respectively. Fermentation with *L. salivarius* enhanced the concentration of rutin in extracts, probably by its release from bounded or conjugated forms.

Lactic acid fermentation significantly increases concentrations of active compounds such as phenolic acids, flavonoids, short-chain fatty acids, etc., infermented fruits, vegetables, and legumes, which have proven health benefits [64]. The profile of compounds during lactic acid fermentation of food and beverages is strongly strain-dependent [65], and, as far as it is known to the authors, previous results on the biotransformation of polyphenolic compounds by *L. salivarius* ATCC 11741 have not been reported. Filannino et al. [66] demonstrated the ability of *Lactiplantibacillus plantarum* to convert phenolic acids into decarboxylated forms. *Lactiplantibacillus plantarum* POM1 can convert protocatechuic acid to catechol, caffeic acid to vinyl catechol, and *p*-coumaric acid to *p*-vinylphenol, with an efficiency of over 90% [66]. Tang et al. [67] demonstrated the conversion of cyanidin-3-O-glucoside to cyanidin and quercetin-3-O-rhamnoside to quercetin during mulberry pomace fermentation using *Lactiplantibacillus plantarum* CICC 20265. Results of this study suggest that fermentation with *L. salivarius* could be a valuable strategy to increase the concentration of polyphenolics of nettle by-products, resulting in extracts with substantial added value. The kinetics of *L. salivarius* growth correspond well with the production and increase in the concentration of specific bioactive compounds in fermented extracts (Figure 1a, Figure 3 and Figure 4). The biotransformation of phenolic compounds occurs during the exponential growth phase of *L. salivarius*, primarily within the first 20 h of fermentation, and these conclusions are also supported by the DPPH, ABTS and TPC assays for most samples.

The decomposition of tannins and lignans, as well as the biotransformation of flavonoids and phenolic acids, can explain the observed positive influence of fermentation on the concentration of phenolic compounds. Bacteria from the former genus *Lactobacillus* can produce glucosidase, esterase, phenolic acid decarboxylase, phenolic acid reductase, and tannase, enzymes involved in the degradation and biotransformation of phenolic compounds in plant extracts [68,69,70,71,72,73]. *L. salivarius* can metabolize lignans [74,75], while tannins do not affect their growth [76]. There are reports for tannase production and metabolism of tannins by other *Lactobacillus* sp. [77]; however, to the best of our knowledge, genes encoding tannase production in *L. salivarius* have not been reported [74,75,76,78]. Different types of glucosyltransferase enzymes are involved in the biosynthesis of rutin, chlorogenic acid, and other glucosides and phenolic acid derivatives in plants [79,80]. They also play an important role in exopolysaccharide biosynthesis and complex carbohydrate metabolism in *L. salivarius* [81,82,83]. Therefore, glycosyltransferases could potentially catalyze the esterification of quercetin, caffeic acid, and *p*-coumaric acid, resulting in an increase in the concentration of rutin and phenolic acid derivatives in the fermented nettle extracts. Klingel et al. [84] proved glycosidation of flavonoids and their glycosides (quercetin and quercetin glycosides, cyanidin-3-O-β-glucoside, naringin and neohesperidin dihydrochalcone, epigallocatechin gallate, and dihydromyricetin) by mutant dextransucrase (glucosyltransferase) from *Lactobacillus reuteri* TMW 1.106. Biotransformation is unlikely to involve decarboxylation [85], as phenolic acid decarboxylase has not been reported for *L. salivarius* so far.

### 3.4. The Influence of Fermentation on Protein Concentration and α-Amino Nitrogen

**Figure 5:**
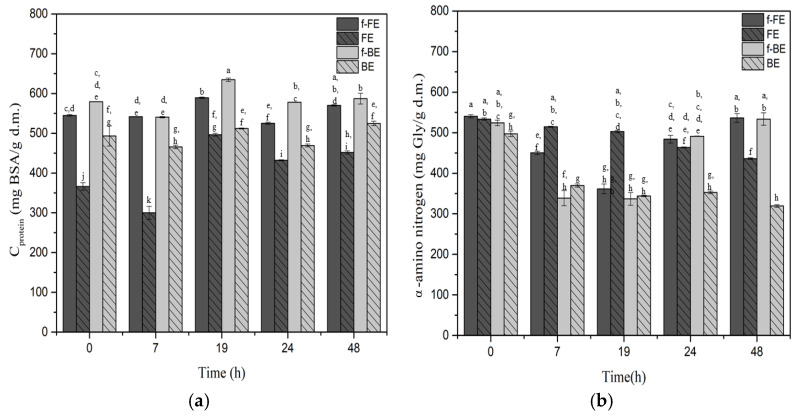
Impact of fermentation on the (**a**) protein concentrations and (**b**) α-amino nitrogen in fermented FE and BE. Symbols: f-FE (fermented common nettle flower extract—dark gray), FE control (unfermented common nettle flower extract—dark gray patterned), f-BE (fermented common nettle by-product extract—light gray), and BE control (unfermented nettle by-product extract—light gray patterned). Different letters above the bars indicate significant differences (*p* < 0.05) among all samples, as determined by two-way ANOVA followed by Tukey’s post hoc test.

During fermentation, LAB grow, increasing the microbial biomass yield. These bacteria contain proteins, and some proteins are also released by bacteria, e.g., enzymes, which may slightly increase the overall protein concentration of the fermented extracts. The influence of fermentation on the protein concentration and α-amino nitrogen in f-FE and f-BE is shown in Figure 5.

Throughout fermentation, the protein concentration increases in both fermented extracts. The highest protein concentration was observed after 19 h of fermentation for both f-BE (589.14 mg BSA/g d.m.) and f-FE (634.63 mg BSA/g d.m.) (Figure 5). The fermented extracts showed a higher protein concentration than the respective controls throughout the entire fermentation. The highest increase in protein concentration, of 44.63% over control, was recorded at 7 h of fermentation in f-BE. The slight increase in protein content observed in the control samples can be attributed to the enhanced solubility of proteins already present in nettle extracts at an elevated incubation temperature (37 °C).

In fermented samples, α-amino nitrogen content was high at the beginning, decreased at intermediate time points, and increased again at 24 h, reflecting dynamic changes driven by the metabolic activity of *L. salivarius*. No significant differences were observed between the initial and final values. However, fermented samples still showed higher α-amino nitrogen concentrations at the end of fermentation compared to the non-fermented ones. The decrease in α-amino nitrogen content, with an increase in protein concentration, can be explained by microbial transformations during fermentation. The free amino acids, which contribute to α-amino nitrogen, are consumed by the microorganism for the synthesis of new microbial proteins or peptides, leading to a decrease in measurable free amino groups [86]. At the same time, fermentation increases the amount of soluble protein detected by the Lowry method. These findings are very suggestive; however, further investigation through specific follow-up studies could elucidate more mechanisms involved and their significance for future applications.

It is important to note that protein concentration is measured in fermented cell-free extracts, indicating that the increase in total protein concentration is likely related to enzyme production, intense metabolic activity in general, and possibly cell lysis later in the process. The remaining cellular biomass of *L. salivarius* can be used to produce pharmaceutical dietary supplements or single-cell proteins. Single-cell proteins or microbial proteins are edible proteins from microorganisms such as bacteria, fungi, yeast, and algae with GRAS status, which are valued for use in food and feed [87], and are also important in the form of postbiotics. It was previously reported that lactic acid fermentation can be used for the production of single-cell proteins [88]. Additionally, common nettle is a valuable source of proteins, containing essential amino acids [89,90]. LAB, including *L. salivarius*, can hydrolyze milk proteins and transform them into bioactive peptides [91,92]. The natural habitat of *L. salivarius* is the oral cavity, and it is commonly used as a probiotic for intraoral application [93,94]. Biotransformation of nettle and similar plant proteins in fermentation by LAB is an additional aspect to be examined in future research. Remaining viable *L. salivarius* is an additional value generated in the process and could be used as an additive, a viable biomass with probiotic potential.

In contrast to synthetic antioxidants and food preservatives, which can induce gut microbiota dysbiosis and could negatively influence human health [95,96], fermented herbal extracts, fortified with probiotic biomass, could be a great alternative. Synthetic antioxidants conventionally used in the past have been linked to skin allergies and gastrointestinal issues [97,98,99,100]. This study proposed a new synbiotic concept: *L. salivarius*-fortified nettle extracts for functional food and dietary supplements. Due to the presence of anti-aging ingredients such as antioxidants and lactic acid, which acts as an exfoliating agent, fermented common nettle extract could also be used in cosmetics [19].

The experimental results for fermented common nettle extracts obtained in this study could also be an important step in producing postbiotic formulations with both antioxidant and other beneficial properties originating from microbial biomass for food and pharmaceutical applications. Fermented extracts could be used as postbiotics if the *L. salivarius* biomass is not preserved in a viable form, for example, at some later stage of processing after fermentation.

The fermented extracts of nettle by-products demonstrated exceptional potential for industrial application due to the significant increase in specific antioxidant compounds during fermentation, robust growth of *L. salivarius*, and increased lactic acid and protein concentrations, upgrading nettle tea by-products as equivalent, or in some cases superior to premium raw materials, such as nettle flowers. Future research should clarify the metabolic pathways and key enzymes involved in *L. salivarius*-driven biotransformation, assess the safety and stability of fermented extracts, and perform pilot-scale trials to verify scalability and industrial applicability. These steps are essential in order to translate laboratory results into commercially viable synbiotic or postbiotic bioactive products.

## 4. Conclusions

This study showed that the fermentation of nettle extracts with *L. salivarius* ATCC 11741 significantly improved the antioxidant compound’s profile, increasing the concentrations of chlorogenic acid, caffeic acid derivatives, rutin, and other bioactive phenols. Nettle by-products are an excellent substrate for *L. salivarius* ATCC 11741 and biotransformation by probiotics, supporting the growth of probiotic bacteria and lactic acid production. The process was carried out in green aqueous media using low-energy ultrasound treatment, in line with the goals of sustainable production and the principles of the circular bioeconomy. Compared to nettle flowers, fermented by-products showed equal or even superior performance in some key parameters, highlighting them as valuable raw materials. The fermentation process not only recovered but also significantly improved the functionality of nettle-derived compounds, as natural antioxidants, food additives, and cosmetic ingredients. These findings confirm the feasibility of the valorization of medicinal plant residues through biotechnology and open new avenues for the development of synbiotic or postbiotic bioactive formulations. Further investigation of the mechanistic pathways of biotransformation by *L. salivarius*, as well as the scalability of this process, could accelerate the integration of fermented plant residues into commercially viable functional products.

## Figures and Tables

**Table 1:** Extract characteristics of inoculated (f-FE, f-BE) and uninoculated (FE, BE) extracts before incubation.

	Dry Matter (g/mL)	pH	DPPH (mM TE/g d.m.)	ABTS (mM TE/g d.m.)	TPC (mM GAE/g d.m.)	Reduced Sugar Content (g GLC/l)	Protein Content (mg BSA/g d.m.)	α-Amino Nitrogen (mg Gly/g d.m.)
**f-FE**	0.089 ± 0.01 ^a^	6.93 ± 0.01 ^b^	207.4 ± 1.4 ^a^	287.8 ± 2.9 ^b^	20.8 ± 0.5 ^ab^	1.1 ± 0.1 ^a^	579.2 ± 0.1 ^a^	524.2 ± 6.7 ^a^
**FE**	0.088 ± 0.01 ^a^	7.01 ± 0.01 ^b^	204.5 ± 4.9 ^a^	291.5 ± 4.4 ^b^	19.8 ± 0.4 ^a^	1.1 ± 0.1 ^a^	493.2 ± 25.2 ^ab^	497.3 ± 6.7 ^b^
**f-BE**	0.093 ± 0.01 ^a^	7.16 ± 0.02 ^a^	154.8 ± 4.2 ^b^	269.5 ± 0.9 ^a^	22.1 ± 0.3 ^b^	1.2 ± 0.1 ^b^	545.0 ± 1.8 ^b^	540.3 ± 4.0 ^a^
**BE**	0.098 ± 0.01 ^a^	7.11 ± 0.03 ^a^	156.964 ± 2.3 ^b^	268.5 ± 3.0 ^a^	25.9 ± 1.0 ^c^	1.2 ± 0.1 ^b^	366.2 ± 9.5 ^c^	533.6 ± 2.7 ^a^

Note: Values are expressed as mean ± standard deviation (n = 2 biological replicates × 2 technical replicates). Different superscript letters within a column indicate statistically significant differences according to one-way ANOVA followed by Tukey’s post hoc test (*p* < 0.05). Abbreviations: TE—Trolox equivalents; GAE—gallic acid equivalents; GLC—glucose; BSA—bovine serum albumin; Gly—glycine; f-FE—fermented flower extract; FE—unfermented flower extract; f-BE—fermented by-product extract; BE—unfermented by-product extract.

## Data Availability

The datasets generated and analyzed during the current study are not publicly available but are available from the corresponding author on reasonable request.

## References

[1] Saha A., Basak B.B. (2020). Scope of Value Addition and Utilization of Residual Biomass from Medicinal and Aromatic Plants. Ind. Crops Prod..

[2] Chandra P., Sharma V. (2019). Marketing Information System and Strategies for Sustainable and Competitive Medicinal and Aromatic Plants Trade. Inf. Dev..

[3] Wei L.S., Goh K.W., Abdul Hamid N.K., Abdul Kari Z., Wee W., Van Doan H. (2022). A Mini-Review on Co-Supplementation of Probiotics and Medicinal Herbs: Application in Aquaculture. Front. Vet. Sci..

[4] Mandpe A., Bhattacharya A., Paliya S., Pratap V., Hussain A., Kumar S. (2022). Life-Cycle Assessment Approach for Municipal Solid Waste Management System of Delhi City. Environ. Res..

[5] Chavan D., Manjunatha G.S., Singh D., Periyaswami L., Kumar S., Kumar R. (2022). Estimation of Spontaneous Waste Ignition Time for Prevention and Control of Landfill Fire. Waste Manag..

[6] Ramachandra T.V., Bharath H.A., Kulkarni G., Han S.S. (2018). Municipal Solid Waste: Generation, Composition and GHG Emissions in Bangalore, India. Renew. Sustain. Energy Rev..

[7] Freitas L.C., Barbosa J.R., da Costa A.L.C., Bezerra F.W.F., Pinto R.H.H., de Carvalho Junior R.N. (2021). From Waste to Sustainable Industry: How Can Agro-Industrial Wastes Help in the Development of New Products?. Resour. Conserv. Recycl..

[8] Khan S., Anjum R., Raza S.T., Ahmed Bazai N., Ihtisham M. (2022). Technologies for Municipal Solid Waste Management: Current Status, Challenges, and Future Perspectives. Chemosphere.

[9] UN—Sustainable Development (2015) The 17 Goals. https://sdgs.un.org/goals.

[10] Venkatesh G. (2022). Circular Bio-Economy—Paradigm for the Future: Systematic Review of Scientific Journal Publications from 2015 to 2021. Circ. Econ. Sustain..

[11] Ahmad T., Esposito F., Cirillo T. (2024). Valorization of Agro-Food by-Products: Advancing Sustainability and Sustainable Development Goals 2030 through Functional Compounds Recovery. Food Biosci..

[12] Toplicean I.-M., Ianuș R.-D., Datcu A.-D. (2024). An Overview on Nettle Studies, Compounds, Processing and the Relation with Circular Bioeconomy. Plants.

[13] Kregiel D., Pawlikowska E., Antolak H. (2018). *Urtica* spp.: Ordinary Plants with Extraordinary Properties. Molecules.

[14] Devkota H.P., Paudel K.R., Khanal S., Baral A., Panth N., Adhikari-Devkota A., Jha N.K., Das N., Singh S.K., Chellappan D.K. (2022). Stinging Nettle (*Urtica dioica* L.): Nutritional Composition, Bioactive Compounds, and Food Functional Properties. Molecules.

[15] Đurović S., Kojić I., Radić D., Smyatskaya Y.A., Bazarnova J.G., Filip S., Tosti T. (2024). Chemical Constituents of Stinging Nettle (*Urtica dioica* L.): A Comprehensive Review on Phenolic and Polyphenolic Compounds and Their Bioactivity. Int. J. Mol. Sci..

[16] Rutto L.K., Xu Y., Ramirez E., Brandt M. (2013). Mineral Properties and Dietary Value of Raw and Processed Stinging Nettle (*Urtica dioica* L.). Int. J. Food Sci..

[17] Bağci E. (2002). Fatty Acid Composition of the Aerial Parts of *Urtica dioica* (Stinging Nettle) L. (*Urticaceae*). Biodiversity.

[18] Dar S.A., Yousuf A.R., Ganai F.A., Sharma P., Kumar N., Singh R. (2012). Bioassay Guided Isolation and Identification of Anti-Inflammatory and Anti-Microbial Compounds from *Urtica dioica* L. (*Urticaceae*) Leaves. Afr. J. Biotechnol..

[19] Bourgeois C., Leclerc É.A., Corbin C., Doussot J., Serrano V., Vanier J.-R., Seigneuret J.-M., Auguin D., Pichon C., Lainé É. (2016). Nettle (*Urtica dioica* L.) as a Source of Antioxidant and Anti-Aging Phytochemicals for Cosmetic Applications. Comptes Rendus Chim..

[20] Li Z., Kanwal R., Yue X., Li M., Xie A. (2024). Polyphenols and Intestinal Microorganisms: A Review of Their Interactions and Effects on Human Health. Food Biosci..

[21] Chrubasik J.E., Roufogalis B.D., Wagner H., Chrubasik S.A. (2007). A Comprehensive Review on Nettle Effect and Efficacy Profiles, Part I: Herba *Urticae*. Phytomedicine.

[22] Stinging Nettle Root Extract Market. https://www.factmr.com/report/stinging-nettle-root-extract-market.

[23] Atwi-Ghaddar S., Zerwette L., Destandau E., Lesellier E. (2023). Supercritical Fluid Extraction (SFE) of Polar Compounds from *Camellia sinensis* Leaves: Use of Ethanol/Water as a Green Polarity Modifier. Molecules.

[24] Sarita B., Samadhan D., Hassan M.Z., Kovaleva E.G. (2025). A Comprehensive Review of Probiotics and Human Health-Current Prospective and Applications. Front. Microbiol..

[25] Sionek B., Szydłowska A., Küçükgöz K., Kołożyn-Krajewska D. (2023). Traditional and New Microorganisms in Lactic Acid Fermentation of Food. Fermentation.

[26] Tiwari S., Kavitake D., Suryavanshi M.V., Shah I.A., Devi P.B., Reddy G.B., Shetty P.H. (2024). An Expanding Frontier in Prebiotic Research and Synbiotic Functional Food Development through Exopolysaccharides from Probiotic Bacteria. Food Humanit..

[27] Rahbar Saadat Y., Yari Khosroushahi A., Pourghassem Gargari B. (2019). A Comprehensive Review of Anticancer, Immunomodulatory and Health Beneficial Effects of the Lactic Acid Bacteria Exopolysaccharides. Carbohydr. Polym..

[28] Ibrahim A.Y., Youness E.R., Mahmoud M.G., Asker M.S., El-Newary S.A. (2020). Acidic Exopolysaccharide Produced from Marine *Bacillus amyloliquefaciens* 3MS 2017 for the Protection and Treatment of Breast Cancer. Breast Cancer Basic Clin. Res..

[29] Darbandi A., Asadi A., Mahdizade Ari M., Ohadi E., Talebi M., Halaj Zadeh M., Darb Emamie A., Ghanavati R., Kakanj M. (2022). Bacteriocins: Properties and Potential Use as Antimicrobials. J. Clin. Lab. Anal..

[30] Wang C., Chang T., Yang H., Cui M. (2015). Antibacterial Mechanism of Lactic Acid on Physiological and Morphological Properties of *Salmonella enteritidis*, *Escherichia coli* and *Listeria monocytogenes*. Food Control.

[31] Li X., Shimizu Y., Kimura I. (2017). Gut Microbial Metabolite Short-Chain Fatty Acids and Obesity. Biosci. Microbiota Food Health.

[32] Fernández J., Redondo-Blanco S., Gutiérrez-del-Río I., Miguélez E.M., Villar C.J., Lombó F. (2016). Colon Microbiota Fermentation of Dietary Prebiotics towards Short-Chain Fatty Acids and Their Roles as Anti-Inflammatory and Antitumour Agents: A Review. J. Funct. Foods.

[33] Chen X.-F., Chen X., Tang X. (2020). Short-Chain Fatty Acid, Acylation and Cardiovascular Diseases. Clin. Sci..

[34] Gaur G., Gänzle M.G. (2023). Conversion of (Poly) Phenolic Compounds in Food Fermentations by Lactic Acid Bacteria: Novel Insights into Metabolic Pathways and Functional Metabolites. Curr. Res. Food Sci..

[35] Zhao Y., Zhong X., Yan J., Sun C., Zhao X., Wang X. (2022). Potential Roles of Gut Microbes in Biotransformation of Natural Products: An Overview. Front. Microbiol..

[36] Zheng J., Wittouck S., Salvetti E., Franz C.M.A.P., Harris H.M.B., Mattarelli P., O’toole P.W., Pot B., Vandamme P., Walter J. (2020). A Taxonomic Note on the Genus *Lactobacillus*: Description of 23 Novel Genera, Emended Description of the Genus *Lactobacillus* Beijerinck 1901, and Union of *Lactobacillaceae* and *Leuconostocaceae*. Int. J. Syst. Evol. Microbiol..

[37] Marco M.L., Heeney D., Binda S., Cifelli C.J., Cotter P.D., Foligné B., Gänzle M., Kort R., Pasin G., Pihlanto A. (2017). Health Benefits of Fermented Foods: Microbiota and Beyond. Curr. Opin. Biotechnol..

[38] Mladenović M., Bogdanović M., Mladenović D., Vuković A.D., Mojović L. (2023). Common Nettle Processing Residues as a Valuable Source of Antioxidants. Proceedings of the 10th International Conference on Sustainable Solid Waste Management.

[39] Koch R. Über Die Neuen Untersuchungsmethoden Zum Nachweis Der Mikrokosmen in Boden, Luft Und Wasser. http://edoc.rki.de/documents/rk/508-274-284/PDF/274-284.pdf.

[40] Negrulescu A., Patrulea V., Mincea M.M., Ionascu C., Vlad-Oros B.A., Ostafe V. (2012). Adapting the Reducing Sugars Method with Dinitrosalicylic Acid to Microtiter Plates and Microwave Heating. J. Braz. Chem. Soc..

[41] Brand-Williams W., Cuvelier M.-E., Berset C. (1995). Use of a Free Radical Method to Evaluate Antioxidant Activity. LWT-Food Sci. Technol..

[42] Re R., Pellegrini N., Proteggente A., Pannala A., Yang M., Rice-Evans C. (1999). Antioxidant Activity Applying an Improved ABTS Radical Cation Decolorization Assay. Free Radic. Biol. Med..

[43] Swain T., Hillis W.E. (1959). The Phenolic Constituents of Prunus Domestica. I.—The Quantitative Analysis of Phenolic Constituents. J. Sci. Food Agric..

[44] Martinović M., Krgović N., Nešić I., Žugić A., Tadić V.M. (2022). Conventional vs. Green Extraction Using Natural Deep Eutectic Solvents—Differences in the Composition of Soluble Unbound Phenolic Compounds and Antioxidant Activity. Antioxidants.

[45] Hartree E.F. (1972). Determination of Protein: A Modification of the Lowry Method That Gives a Linear Photometric Response. Anal. Biochem..

[46] Lie S. (1973). The ebc-ninhydrin method for determination of free alpha amino nitrogen. J. Inst. Brew..

[47] Corry J.E.L., Curtis G.D.W., Baird R.M. (2003). De Man Rogosa and Sharpe (MRS) Agar. Handbook of Culture Media for Food Microbiology.

[48] Molino S., Lerma-Aguilera A., Jiménez-Hernández N., Gosalbes M.J., Rufián-Henares J.Á., Francino M.P. (2021). Enrichment of Food with Tannin Extracts Promotes Healthy Changes in the Human Gut Microbiota. Front. Microbiol..

[49] Taheri Y., Quispe C., Herrera-Bravo J., Sharifi-Rad J., Ezzat S.M., Merghany R.M., Shaheen S., Azmi L., Prakash Mishra A., Sener B. (2022). *Urtica dioica*-Derived Phytochemicals for Pharmacological and Therapeutic Applications. Evid. Based. Complement. Alternat. Med..

[50] Li Y., Raftis E., Canchaya C., Fitzgerald G.F., van Sinderen D., O’Toole P.W. (2006). Polyphasic Analysis Indicates That *Lactobacillus salivarius* subsp. Salivarius and Lactobacillus salivarius subsp. salicinius Do Not Merit Separate Subspecies Status. Int. J. Syst. Evol. Microbiol..

[51] Śliżewska K., Chlebicz-Wójcik A. (2020). Growth Kinetics of Probiotic *Lactobacillus* Strains in the Alternative, Cost-Efficient Semi-Solid Fermentation Medium. Biology.

[52] Popov S., Skeledžija S., Šorgić S., Zeković Z., Micić D., Radulović A., Đurović S. (2020). Application of Contemporary Extraction Techniques for Elements and Minerals Recovery from Stinging Nettle Leaves. Appl. Sci..

[53] Hou F., Su T., Chen Y., Dong L., Zhang M., Huang F. (2025). Novel Insights into Bound Phenolics: Conversion and Release of Phenolic Compounds in Lychee Pulp by Heat Pump Drying and Lactic Acid Bacterial Fermentation. Food Res. Int..

[54] Khare V., Kushwaha P., Verma S., Gupta A., Srivastava S., Rawat A.K.S. (2012). Pharmacognostic Evaluation and Antioxidant Activity of *Urtica dioica* L. *Chin*. Med..

[55] Güder A., Korkmaz H. (2012). Evaluation of In-Vitro Antioxidant Properties of Hydroalcoholic Solution Extracts *Urtica dioica* L., Malva Neglecta Wallr. and Their Mixture. Iran. J. Pharm. Res. IJPR.

[56] Carvalho A.R., Costa G., Figueirinha A., Liberal J., Prior J.A.V., Lopes M.C., Cruz M.T., Batista M.T. (2017). *Urtica* spp.: Phenolic Composition, Safety, Antioxidant and Anti-Inflammatory Activities. Food Res. Int..

[57] Vajić U.-J., Grujić-Milanović J., Živković J., Šavikin K., Gođevac D., Miloradović Z., Bugarski B., Mihailović-Stanojević N. (2015). Optimization of Extraction of Stinging Nettle Leaf Phenolic Compounds Using Response Surface Methodology. Ind. Crops Prod..

[58] Gan R., Shah N.P., Wang M., Lui W., Corke H. (2017). *Lactobacillus plantarum* WCFS1 Fermentation Differentially Affects Antioxidant Capacity and Polyphenol Content in Mung Bean (*Vigna radiata*) and Soya Bean (*Glycine max*) Milks. J. Food Process. Preserv..

[59] Wianowska D., Gil M. (2019). Recent Advances in Extraction and Analysis Procedures of Natural Chlorogenic Acids. Phytochem. Rev..

[60] Orčić D., Francišković M., Bekvalac K., Svirčev E., Beara I., Lesjak M., Mimica-Dukić N. (2014). Quantitative Determination of Plant Phenolics in *Urtica dioica* Extracts by High-Performance Liquid Chromatography Coupled with Tandem Mass Spectrometric Detection. Food Chem..

[61] Hernández T., Estrella I., Pérez-Gordo M., Alegría E.G., Tenorio C., Ruiz-Larrrea F., Moreno-Arribas M.V. (2007). Contribution of Malolactic Fermentation by *Oenococcus Oeni* and *Lactobacillus Plantarum* to the Changes in the Nonanthocyanin Polyphenolic Composition of Red Wine. J. Agric. Food Chem..

[62] Fritsch C., Heinrich V., Vogel R.F., Toelstede S. (2016). Phenolic Acid Degradation Potential and Growth Behavior of Lactic Acid Bacteria in Sunflower Substrates. Food Microbiol..

[63] Zeković Z., Cvetanović A., Švarc-Gajić J., Gorjanović S., Sužnjević D., Mašković P., Savić S., Radojković M., Đurović S. (2017). Chemical and Biological Screening of Stinging Nettle Leaves Extracts Obtained by Modern Extraction Techniques. Ind. Crops Prod..

[64] Deveci G., Çelik E., Ağagündüz D., Bartkiene E., Rocha J.M.F., Özogul F. (2023). Certain Fermented Foods and Their Possible Health Effects with a Focus on Bioactive Compounds and Microorganisms. Fermentation.

[65] Huang S., Vignolles M.L., Chen X.D., Le Loir Y., Jan G., Schuck P., Jeantet R. (2017). Spray Drying of Probiotics and Other Food-Grade Bacteria: A Review. Trends Food Sci. Technol..

[66] Filannino P., Bai Y., Di Cagno R., Gobbetti M., Gänzle M.G. (2015). Metabolism of Phenolic Compounds by *Lactobacillus* spp. during Fermentation of Cherry Juice and Broccoli Puree. Food Microbiol..

[67] Tang S., Cheng Y., Wu T., Hu F., Pan S., Xu X. (2021). Effect of *Lactobacillus plantarum*-Fermented Mulberry Pomace on Antioxidant Properties and Fecal Microbial Community. LWT.

[68] Jiménez N., Esteban-Torres M., Mancheño J.M., de las Rivas B., Muñoz R. (2014). Tannin Degradation by a Novel Tannase Enzyme Present in Some *Lactobacillus plantarum* Strains. Appl. Environ. Microbiol..

[69] Bhat R., Suryanarayana L.C., Chandrashekara K.A., Krishnan P., Kush A., Ravikumar P. (2015). *Lactobacillus plantarum* Mediated Fermentation of *Psidium guajava* L. Fruit Extract. J. Biosci. Bioeng..

[70] Sabokbar N., Khodaiyan F. (2016). Total Phenolic Content and Antioxidant Activities of Pomegranate Juice and Whey Based Novel Beverage Fermented by Kefir Grains. J. Food Sci. Technol..

[71] Li Z., Teng J., Lyu Y., Hu X., Zhao Y., Wang M. (2018). Enhanced Antioxidant Activity for Apple Juice Fermented with *Lactobacillus plantarum* ATCC14917. Molecules.

[72] Adebo O.A., Gabriela Medina-Meza I. (2020). Impact of Fermentation on the Phenolic Compounds and Antioxidant Activity of Whole Cereal Grains: A Mini Review. Molecules.

[73] Zhou Y., Wang R., Zhang Y., Yang Y., Sun X., Zhang Q., Yang N. (2020). Biotransformation of Phenolics and Metabolites and the Change in Antioxidant Activity in Kiwifruit Induced by *Lactobacillus plantarum* Fermentation. J. Sci. Food Agric..

[74] Bravo D., Peirotén Á., Álvarez I., Landete J.M. (2017). Phytoestrogen Metabolism by Lactic Acid Bacteria: Enterolignan Production by *Lactobacillus Salivarius* and *Lactobacillus Gasseri* Strains. J. Funct. Foods.

[75] de la Bastida A.R., Peirotén Á., Langa S., Álvarez I., Arqués J.L., Landete J.M. (2021). Metabolism of Flavonoids and Lignans by *Lactobacilli* and *Bifidobacteria* Strains Improves the Nutritional Properties of Flaxseed-Enriched Beverages. Food Res. Int..

[76] Girard M., Bee G. (2020). Invited Review: Tannins as a Potential Alternative to Antibiotics to Prevent Coliform Diarrhea in Weaned Pigs. Animal.

[77] Ueda S., Nomoto R., Yoshida K., Osawa R. (2014). Comparison of Three Tannases Cloned from Closely Related *Lactobacillus* Species: *L. Plantarum*, *L. Paraplantarum*, and *L. Pentosus*. BMC Microbiol..

[78] Kamijo M., Kanazawa T., Funaki M., Nishizawa M., Yamagishi T. (2008). Effects of Rosa Rugosa Petals on Intestinal Bacteria. Biosci. Biotechnol. Biochem..

[79] Kianersi F., Abdollahi M.R., Mirzaie-Asl A., Dastan D., Rasheed F. (2020). Identification and Tissue-Specific Expression of Rutin Biosynthetic Pathway Genes in Capparis Spinosa Elicited with Salicylic Acid and Methyl Jasmonate. Sci. Rep..

[80] Aksamit-Stachurska A., Korobczak-Sosna A., Kulma A., Szopa J. (2008). Glycosyltransferase Efficiently Controls Phenylpropanoid Pathway. BMC Biotechnol..

[81] Yang J., Shang P., Zhang B., Wang J., Du Z., Wang S., Xing J., Zhang H. (2023). Genomic and Metabonomic Methods Reveal the Probiotic Functions of Swine-Derived *Ligilactobacillus salivarius*. BMC Microbiol..

[82] Oleksy M., Klewicka E. (2018). Exopolysaccharides Produced by *Lactobacillus* sp.: Biosynthesis and Applications. Crit. Rev. Food Sci. Nutr..

[83] Rajoka M.S.R., Wu Y., Mehwish H.M., Bansal M., Zhao L. (2020). *Lactobacillus* Exopolysaccharides: New Perspectives on Engineering Strategies, Physiochemical Functions, and Immunomodulatory Effects on Host Health. Trends Food Sci. Technol..

[84] Klingel T., Hadamjetz M., Fischer A., Wefers D. (2019). Glucosylation of Flavonoids and Flavonoid Glycosides by Mutant Dextransucrase from *Lactobacillus reuteri* TMW 1.106. Carbohydr. Res..

[85] López de Felipe F. (2023). Revised Aspects into the Molecular Bases of Hydroxycinnamic Acid Metabolism in *Lactobacilli*. Antioxidants.

[86] Schaan K., Hughes P. (2024). A Comparison of Free Amino Nitrogen and Yeast-Assimilable Nitrogen Measurement Methods for Use in Alcoholic Fermentation of Whey. J. Dairy Sci..

[87] Li Y.P., Ahmadi F., Kariman K., Lackner M. (2024). Recent Advances and Challenges in Single Cell Protein (SCP) Technologies for Food and Feed Production. npj Sci. Food.

[88] Murali Sankar P., Karthiba L., Shreedevasena S., Anantha Raju P., Vanitha S., Salama E.A.A., Kamalakannan A., Jeyakumar P. (2023). Bacterial Single Cell Protein: Applications, Productions, and Commercialization: Opportunities and Challenges. Food Microbiology Based Entrepreneurship.

[89] Kronbauer M., Shorstkii I., Botelho da Silva S., Toepfl S., Lammerskitten A., Siemer C. (2023). Pulsed Electric Field Assisted Extraction of Soluble Proteins from Nettle Leaves ( *Urtica dioica* L.): Kinetics and Optimization Using Temperature and Specific Energy. Sustain. Food Technol..

[90] Adhikari B.M., Bajracharya A., Shrestha A.K. (2016). Comparison of Nutritional Properties of Stinging Nettle (*Urtica dioica*) Flour with Wheat and Barley Flours. Food Sci. Nutr..

[91] Ahtesh F.B., Stojanovska L., Apostolopoulos V. (2018). Anti-Hypertensive Peptides Released from Milk Proteins by Probiotics. Maturitas.

[92] Eiteman M.A., Ramalingam S. (2015). Microbial Production of Lactic Acid. Biotechnol. Lett..

[93] Kucia M., Wietrak E., Szymczak M., Kowalczyk P. (2020). Effect of *Ligilactobacillus salivarius* and Other Natural Components against Anaerobic Periodontal Bacteria. Molecules.

[94] Bogdanović M., Mladenović D., Mojović L., Djuriš J., Djukić-Vuković A. (2024). Intraoral Administration of Probiotics and Postbiotics: An Overview of Microorganisms and Formulation Strategies. Braz. J. Pharm. Sci..

[95] Liu C., Zhan S., Tian Z., Li N., Li T., Wu D., Zeng Z., Zhuang X. (2022). Food Additives Associated with Gut Microbiota Alterations in Inflammatory Bowel Disease: Friends or Enemies?. Nutrients.

[96] Zhou X., Qiao K., Wu H., Zhang Y. (2023). The Impact of Food Additives on the Abundance and Composition of Gut Microbiota. Molecules.

[97] Jeong S.-H., Kim B.-Y., Kang H.-G., Ku H.-O., Cho J.-H. (2005). Effects of Butylated Hydroxyanisole on the Development and Functions of Reproductive System in Rats. Toxicology.

[98] Engin A.B., Bukan N., Kurukahvecioglu O., Memis L., Engin A. (2011). Effect of Butylated Hydroxytoluene (E321) Pretreatment versus l-Arginine on Liver Injury after Sub-Lethal Dose of Endotoxin Administration. Environ. Toxicol. Pharmacol..

[99] Botterweck A.A., Verhagen H., Goldbohm R., Kleinjans J., van den Brandt P. (2000). Intake of Butylated Hydroxyanisole and Butylated Hydroxytoluene and Stomach Cancer Risk: Results from Analyses in the Netherlands Cohort Study. Food Chem. Toxicol..

[100] Randhawa S., Bahna S.L. (2009). Hypersensitivity Reactions to Food Additives. Curr. Opin. Allergy Clin. Immunol..

